# Evaluation of an Intravitreal Rho-Associated Kinase Inhibitor Depot Formulation in a Rat Model of Diabetic Retinopathy

**DOI:** 10.3390/pharmaceutics13081105

**Published:** 2021-07-21

**Authors:** Cecile Lebon, Heike Neubauer, Marianne Berdugo, Kimberley Delaunay, Elke Markert, Kolja Becker, Katja S. Baum-Kroker, Jürgen Prestle, Holger Fuchs, Remko A. Bakker, Francine Behar-Cohen

**Affiliations:** 1 Team 17: Physiopathology of Ocular Diseases: Therapeutic Innovations, Centre de Recherche des Cordeliers, Sorbonne Université, Inserm, Université de Paris, F-75006 Paris, France; cecile.lebon@sorbonne-universite.fr (C.L.); marianne.berdugo@gmail.com (M.B.); kimberley.delaunay@sorbonne-universite.fr (K.D.); 2 CardioMetabolic Diseases Research, Boehringer Ingelheim Pharma GmbH & Co KG, D-88397 Biberach, Germany; heike.neubauer@boehringer-ingelheim.com (H.N.); juergen.prestle@boehringer-ingelheim.com (J.P.); holger.fuchs@boehringer-ingelheim.com (H.F.); remko.bakker@boehringer-ingelheim.com (R.A.B.); 3 Global Computational Biology and Digital Sciences, Boehringer Ingelheim Pharma GmbH & Co KG, D-88397 Biberach, Germany; elke.markert@boehringer-ingelheim.com (E.M.); kolja.becker@boehringer-ingelheim.com (K.B.); 4 Drug Discovery Sciences, Boehringer Ingelheim Pharma GmbH & Co KG, D-88397 Biberach, Germany; katja.baum-kroker@boehringer-ingelheim.com; 5 Assistance Publique, Hôpitaux de Paris, Hôpital Cochin, Ophthalmopole, 75014 Paris, France

**Keywords:** diabetic retinopathy, rho-kinase inhibitor, slow-release formulation, retinal pigment epithelium, retinal hypoxia, vasoconstriction, retinal barrier restoration

## Abstract

Rho-associated kinase (ROCK) activation was shown to contribute to microvascular closure, retinal hypoxia, and to retinal pigment epithelium (RPE) barrier disruption in a rat model of diabetic retinopathy. Fasudil, a clinically approved ROCK inhibitor, improved retinal perfusion and reduced edema in this model, indicating that ROCK inhibition could be a promising new therapeutic approach for the treatment of diabetic retinopathy. However, due to its short intravitreal half-life, fasudil is not suitable for long-term treatment. In this study, we evaluated a very potent ROCK1/2 inhibitor (BIRKI) in a depot formulation administered as a single intravitreal injection providing a slow release for at least four weeks. Following BIRKI intravitreal injection in old Goto-Kakizaki (GK) type 2 diabetic rats, we observed a significant reduction in ROCK1 activity in the retinal pigment epithelium/choroid complex after 8 days and relocation of ROCK1 to the cytoplasm and nucleus in retinal pigment epithelium cells after 28 days. The chronic ROCK inhibition by the BIRKI depot formulation restored retinal pigment epithelial cell morphology and distribution, favored retinal capillaries dilation, and reduced hypoxia and inner blood barrier leakage observed in the diabetic retina. No functional or morphological negative effects were observed, indicating suitable tolerability of BIRKI after intravitreous injection. In conclusion, our data suggest that sustained ROCK inhibition, provided by BIRKI slow-release formulation, could be a valuable treatment option for diabetic retinopathy, especially with regard to the improvement of retinal vascular infusion and protection of the outer retinal barrier.

## 1. Introduction

With increasing prevalence and a projected 629 million affected patients by 2045, diabetes presents a major public health concern [1]. More than one-third of diabetic patients develop some degree of diabetic retinopathy [2], and this represents a growing cause of blindness worldwide. Diabetic retinopathy is diagnosed by retinal microvascular lesions detectable on fundus examination, but outer retinal barrier breakdown, reduction in capillary flow, and neurodegeneration precede detectable retinal microangiopathy [3,4]. Current treatments target stages of diabetic retinopathy when vision has already been affected [5], including laser photocoagulation of the peripheral ischemic retina or intraocular injection of anti-VEGF (vascular endothelial growth factor) and corticosteroids to reduce macular edema [6]. Following treatment, major complications may be reduced. However, macular edema and macular ischemia remain major causes of visual impairment, prompting the identification of potential additional molecular regulatory targets to enable the development of adequate treatment options for diabetic retinopathy.

Several studies have shown the potential of Rho-associated kinase (ROCK) inhibitors in the treatment of diabetic retinopathy. ROCK1 and ROCK2 are two downstream effectors of the small GTPase RhoA. Both isoforms regulate junction proteins and play an important role in cell migration by enhancing cell contractility [7]. Furthermore, a role in cellular responses requiring actomyosin contractility, such as smooth muscle contraction, axonal growth, and cell division, has been reported [8]. ROCK1 isoform seems to be predominant in epithelial polarized cells and is involved in endothelial cell shape modification [9,10], while abnormal vascular activation of ROCK1 is involved in hypertension, stroke, and other cardiovascular diseases [11,12].

In diabetes-induced microvascular damages and macular edema, ROCK inhibition has demonstrated promising results [13]. In vitro, in retinal glial Müller cells, the activation of ROCK1 under diabetic conditions up-regulated pro-permeability and inflammatory mediators. These were normalized by the ROCK1/2 inhibitor fasudil [14]. Cell death, migration, and contractility induced by oxidative stress, and hypoxia was normalized by the ROCK pathway inhibitor Y-27632 [15]. In addition to its effect on retractile properties of activated glial cells, the inhibition of ROCK showed clear effects on the retinal vasculature, reducing vasoconstriction and subsequent retinal hypoxia [7,16].

In models of type 1 diabetes induced by streptozotocin administration and in oxygen-induced retinopathy, the intraocular administration of the ROCK inhibitor AMA0428 prevented proliferative retinopathy and pre-retinal neovascularization [17]. In a pilot study including 44 patients with diabetic macular edema, treated monthly with either bevacizumab (anti-VEGF) or with the combination of bevacizumab and fasudil for three consecutive months, fasudil showed an additive effect on macular edema reduction [18]. In a previous study, we demonstrated that in the Goto-Kakizaki (GK) type 2 diabetic model, ROCK1 over-activation induced outer retinal barrier permeability in the retinal pigment epithelium. In the retinal vasculature, ROCK1 activation further induced endothelial cell blebbing and subsequent microvessel occlusion and retinal hypoxia. Due to the short half-life of fasudil, repeated intraocular injections were required to normalize retinal barrier function and retinal perfusion [19]. However, frequent repetition of intraocular injections does not present a suitable treatment option and limits further clinical development.

In this study, we have evaluated the potential of BIRKI (Boehringer Ingelheim Rho kinase inhibitor), a novel and potent inhibitor of ROCK1/2 in the GK diabetic retinopathy model. Based on its innovative physicochemical characteristics, it can be administered as a single intravitreous injection forming a depot in the vitreous. Slow release from the depot maintained sufficient active compound exposure for several weeks.

## 2. Material and Methods

### 2.1. Animals

Nine- to eighteen-month-old male Goto-Kakizaki (GK) rats (Taconic Model for life, Europe distribution, Ejby, Denmark), a Wistar strain of non-obese type 2 diabetes, were used in this study. All experiments in this study were performed in accordance with the Association for Research in Vision and Ophthalmology statement for the use of animals in ophthalmic and vision research. The local ethics committee, European Council Charles Darwin of the University Paris Descartes (authorization numbers 05, ce5/2012/143-03952.03, A75-580) approved all submitted experimental procedures. Animals were housed under a 12 h light/dark cycle with free access to standardized pelleted food and water. Their average weight was 350 g. The diabetic status of GK rats was defined by measurement of the plasma concentration of glycosylated hemoglobin HbA1c (A1C NOW + multitest system, Bayer Healthcare Sunnyvale, Sunnyvale, CA, USA).

The pharmacokinetic profile of BIRKI was studied in 8 male brown Norway (BN/Crl) rats (Charles River Research Models and Services Germany GmbH). All animal experiments were conducted in accordance with the German and European Animal Welfare Act and authorized by the Regierungspräsidium Tübingen as the responsible local German authority under reference numbers 19-020-G (11 September 2019). The animals had an average weight of 220 g (range 204–260 g) and were housed under a 12 h light/dark cycle with free access to standardized pelleted food (Granovit AG) and water.

### 2.2. Pharmacokinetic Analysis

BIRKI was administered by intravitreal injection into each eye of the brown Norway rats at a concentration of 25 mg/mL and a dose volume of 5 µL. The compound formulation was a liquid suspension in a vehicle consisting of 0.9% NaCl and 0.1% polysorbate 80. At each time point (1, 4, 7, 11, and 14 days post-injection), 2 animals were euthanized by anesthetic overdose followed by exsanguination. Immediately following euthanasia, the aqueous and vitreous humor were collected as described in Rimpelä et al. (2020) [20]. In brief, aqueous humor was collected using an insulin syringe (30G) from the anterior chamber. A sample from the vitreous humor was collected via a glass capillary using a modification of the mousetrap technique. Blood samples were obtained under isoflurane anesthesia for all available animals at 0.17, 1, 2, 3, 4, 8, 11, and 14 days post-injection. Blood was collected from the sublingual vein into K3EDTA coated vials (Sarstedt, Nümbrecht, Germany), and plasma was obtained by centrifugation for 5 min at approximately 3500 g. Drug concentrations in the plasma and humor samples were determined by liquid chromatography coupled to tandem mass spectrometry (LC-MS/MS).

### 2.3. Treatment

A total of 39 GK rats were treated with a single intravitreal injection of 3 µL of BIRKI formulation (at concentration 10 mg/mL) or vehicle under general anesthesia (with a mixture of xylazine 4 mg/kg and ketamine 40 mg/kg by intra-muscular injection). Both eyes were treated with the same compound. The control group received the vehicle (0.9% sodium chloride incl. 0.1% polysorbate 80). In order to test for sustained ocular exposure, we evaluated the amount of BIRKI in the posterior segment 8 days (at dissection), 12 days in vivo, and 28 days (at dissection) after the injection.

### 2.4. Evaluation of Retinal Hypoxia and Retinal Vessels

Effects of BIRKI on retinal perfusion and hypoxia were evaluated using a hypoxyprobe-1 kit (Hypoxyprobe, Inc. Burlington, MA, USA). This kit contains pimonidazole, a probe classically used to detect hypoxia. Three hours prior to sacrifice, rats received an intraperitoneal injection of pimonidazole (60 mg/kg body weight). Rats were sacrificed, and the enucleated eyes were fixed in paraformaldehyde 4% for 30 min. The retinas were flat-mounted, fixed 10 min in acetone at −20 °C, blocked with fetal bovine serum 10% in PBS with Triton 0.1% for 30 min, and incubated overnight with anti-pimonidazole antibody (1:100) in blocking solution. After several rinses, the retinas were incubated with secondary antibody (Alexa Fluor conjugated 546 anti-mice, Invitrogen, Waltham, MA, USA) and with FITC-conjugated lectin (Sigma-Aldrich, St. Louis, MI, USA) at a dilution of 1:200. Finally, flat mounts were mounted using fluorescent aqueous mounting medium (Dako Agilent, Santa Clara, CA, USA), and images were acquired with confocal microscopy (LSM 710 Carl Zeiss, Oberkochen, Germany). Measurements were performed using ImageJ software, a macro tool [19] developed on Fiji software, and statistics using GraphPad Prism 8.

### 2.5. Retinal Pigment Epithelium (RPE) Flat Mounts and ROCK Immunohistochemistry

RPE/choroid complexes were obtained according to the protocol described above. After the blocking step, RPE was incubated with anti-ROCK1 primary antibody at a dilution of 1:100 (Santa Cruz Biotechnology, Inc., Dallas, TX, USA) in blocking solution overnight. After rinses, flat mounts were incubated with anti-rabbit secondary antibody at 1:200 (Molecular Probes Alexa Fluor 647), with phalloidin staining (Life Technologies, Carlsbad, CA, USA) at dilution 1:300 for 1 h and with DAPI at dilution 1:5000 for 5 min under constant agitation. Flat mounts were finally mounted with fluorescent aqueous mounting medium (Dako Agilent, Santa Clara, CA, USA). Images were acquired with a confocal microscope (LSM 710 Carl Zeiss, Oberkochen, Germany) and measurements were performed using a macro tool [19] developed on Fiji software and statistics using GraphPad Prism 8.

### 2.6. Electroretinography

To evaluate the negative effects of the BIRKI treatment on retinal function, we recorded electroretinograms (ERG) on 14-month-old GK rats 8 days before the treatment (as baseline recording) and 26 days after the treatment as previously described [20]. Briefly, rats were dark-adapted for 18 h and were anesthetized with a mixture of xylazine (8 mg/kg) and ketamine (80 mg/kg) by intra-muscular injection. A drop of oxybuprocaine (Novesine Novartis Ophthalmics, Basel, Switzerland) was administered to desensitize the corneas, and tropicamide (Novartis Ophthalmics) was used to dilate the pupils. Gold wire ring electrodes were placed on both corneas, and stainless-steel needle electrodes were inserted into the forehead as reference electrodes. A needle electrode was inserted subcutaneously at the tail base for grounding. All these manipulations were performed under dim red light. Full-field ERG using achromatic light stimuli were recorded using the Ganzfeld VisioSystem device (Siem Biomedicale, Nîmes, France): scotopic (essentially rod-driven responses), oscillatory potentials (internal retina response), photopic (essentially cone-driven response), and mixed (whole retina) responses were analyzed for amplitudes (μV) and implicit times (ms), both before and after treatment. Values pre- and post-treatment were compared (a- and b-wave amplitudes and corresponding implicit times), and changes in those parameters were compared between the BIRKI vs. vehicle groups. For scotopic electroretinograms in the dark-adapted state, flash intensities ranged from 0.0003 to 10 cd.s/m². Five flashes of 10 ms per intensity were applied at a frequency of 0.5 Hz for −30 to 0 dB and for 30 ms for 10 cd.s/m² (0 dB). For photopic electroretinograms, following a 5-minute-long rod-suppressing background of 25 cd.s/m², 5 cone-stimulating flashes of 10 cd.s/m² (duration 79 ms) were applied, and responses averaged. Mixed (both rods and cones driven responses) ERGs were performed simultaneously on both eyes, with a 3 cd.s/m² achromatic flash intensity (duration 40 ms), and the amplifier set to 0.5 Hz. Five responses were averaged again. Data are presented as mean ± SEM. Mann–Whitney test was performed, *p* < 0.05 was considered significant.

### 2.7. Blood-Retinal Barrier Evaluation

To evaluate leakage from the blood-retinal barrier breakdown, 200 µL of 150 kDa FITC-dextran at 50 mg/mL diluted in PBS was injected intravenously in the tail of rats prior to the sacrifice. Rats were killed once FITC-dextran was visible in the urine. Eyes were enucleated, mounted in Tissue-Tek OCT, and frozen in liquid nitrogen. Cryosections 10 µm-thick were cut on a microtome (Leica CM3050S, Wetzlar, Germany. Leakage of FITC-dextran and albumin was evaluated by measuring the fluorescence within neuroretinas (vessels excluded). Images were acquired with a fluorescent U-25ND25 Olympus microscope and analyzed with ImageJ software.

### 2.8. Tolerability Evaluation

TUNEL assay was performed on 10 µm-thick cryosections of BIRKI and vehicle-treated rats to evaluate cell death within the retina. Sections were first fixed 15 min in paraformaldehyde 4% and permeabilized 15 min in 0.3% Triton X-100/PBS. The TUNEL assay was performed according to the manufacturer’s instructions (TMR red protocol, Roche diagnostics, Basel Switzerland). Nuclei were stained by a 5 min incubation with DAPI at 1:5000 in PBS, and sections were mounted with fluorescent aqueous mounting medium (Dako Agilent, Santa Clara Ca, USA) Images were acquired with a fluorescent U-25ND25 Olympus microscope, and measurements were performed with ImageJ software.

### 2.9. ROCK Activity

ROCK activity was measured using the ROCK activity Immunoblot kit (Cell Biolabs Inc., San Diego, CA, USA). Rats were sacrificed 8 days after the intravitreal injection of either BIRKI or vehicle. Eyes were enucleated, and both neuroretinas and RPE/choroid complexes were dissected. Proteins were extracted in the lysis buffer from the kit with a pellet pestle motor on ice. The ROCK activity assay was then performed according to the manufacturer’s instructions. Western blots were performed to detect ROCK activity with precast gels and iBlot 2 stacks (Thermo Fisher Scientific, Carlbad, CA, USA). Images were taken with the iBright imaging system (Thermo Fisher Scientific, USA) and quantified with ImageJ software.

### 2.10. RNA Isolation and Quality Control

A total of 28 days after treatment, rats were sacrificed with CO2. After enucleation, eyes were dissected on ice, neuroretinas and RPE/choroid complexes were collected in Eppendorf tubes, snap-frozen in nitrogen immediately, and then stored at −80 °C until the preparation for sequencing. Tissue samples were homogenized on dry ice in 200 µL Qiazol using a micro pestle for 10 s. After homogenization, 500 µL of Qiazol was added, and RNA isolation was performed with miRNeasy Micro Kit (Qiagen) according to manufacturer’s instructions, including an on-column DNase digestion. The quantity and quality of the 24 total RNA samples (12 RPE/Choroid, 12 neuroretinas) was assessed using the fluorescence-based Broad Range Quant-iT RNA Assay Kit (Thermo Fisher) and the Standard Sensitivity RNA Analysis DNF-471 Kit on a 96-channel Fragment Analyzer (Agilent), respectively. While the concentrations averaged at 83.7 ng/µL, the RIN ranged from 6.2 to 9.4.

### 2.11. Transcriptome Profiling with Total RNA Sequencing

Total RNA normalization was performed using the MicroLab STAR automated liquid platform (Hamilton). Input of 100 ng was used for library construction with the NEBNext Ultra II Directional RNA Library Prep Kit for Illumina #E7760, together with the QIAseq FastSelect RNA Removal Kit (Qiagen, Hilden, Germany) upstream and the NEXNext Multiplex Oligos for Illumina #E7600 downstream (all New England Biolabs). Fragmentation time was set to 15 min. A total of 13 PCR cycles was used for the index PCR while the final libraries were eluted in 30 µL. Deviating from the manufacturer’s protocol, AMPure XP beads (Beckman Coulter, Brea, CA, USA) were used at the double-stranded cDNA purification step instead of the recommended SPRIselect beads. Quantification of the total RNA sequencing libraries was performed using the fluorescence dye-based methodology High-Sensitivity dsDNA Quanti-iT Assay Kit (Thermo Fisher) on a Synergy HTX (BioTek, Winooski, VT, USA). Library molarity averaged at 165 nM. Size distribution and adapter dimer presence of the total libraries were assessed by the High-Sensitivity NGS Fragment DNF-474 Kit on a 96-channel Fragment Analyzer (Agilent). Sequencing libraries were normalized on the MicroLab STAR (Hamilton), pooled, and spiked in with PhiX Control v3 (Illumina, San Diego, CA, USA). The library pool was subsequently clustered on an S4 Flow Cell and sequenced on a NovaSeq 6000 Sequencing System (Illumina) with dual index, paired-end reads at 2 × 100 bp length (read parameters: Rd1: 101, Rd2: 8, Rd3: 8, Rd4: 101), reaching an average depth of 66.4 million pass-filter reads per sample (9.0% CV).

### 2.12. Data Processing

FastQ files from the RNA-seq experiment were processed as follows: Filtered reads were mapped against the rattus norvegicus genome Rnor_6.0 (ensemble86) using the STAR aligner software (STAR version 2.5.2b), allowing for soft clipping of adapter sequences. Transcripts were quantified on gene level using the featureCount package (featureCount version 1.5.1). Quality controls were carried out using FastQC (FastQC version 0.11.5), picardmetrics (picardmetrics version 0.2.4), and dupRadar (dupRadar version 1.0.0) at the respective steps.

Due to quality concerns, samples from eyes displaying red coloring were removed from subsequent bioinformatics analysis. Only genes with expression above 64 counts across all remaining 20 samples were kept for further analysis (14.568 genes). Raw counts were normalized by library size (Counts Per Million-CPM) and log-transformed using the voom function provided by the limma R package (version 3.44.3). Initial PCA analysis (pcaMethods version 1.80.0) showed a correlation of the first principal component with technical sample metrics derived from manual RNA QC, Star, fastQC, picardmetrics, or featureCounts. We explicitly corrected for strong confounding effects (absolute Spearman correlation coefficient > 0.8), taking into account the following variables: Material.Conc., RIN, insertion_length, PCT_UTR_BASES, PCT_MRNA_BASES, PCT_USABLE_BASES. In more detail, we designed linear models with CPM expression values as response and confounding variables as covariates (lm function, stats R package version 4.0.2). *t*-test statistics on the covariate were calculated for each linear model to determine its statistical significance. In case the *p*-value for a linear model was below 0.05, the expression values of the gene were replaced by the residuals plus the intercept of the linear model.

Differential expression analysis was carried out on voom normalized expression values using lmFit and eBayes functions provided by the limma package. We converted ensembl gene IDs to official gene symbols using the biomaRt package (version 2.44.4), setting the host parameter to ‘oct2016.archive.ensembl.org’.

Generally Applicable Gene-set Enrichment for Pathway (GAGE) Analysis was performed using the GAGE R package (version 2.38.3). Here we included KEGG, Reactome, and GO Biological Process (GO BP) gene sets provided by MSigDB (version 6.2). The directionality parameter in GAGE was set to same.dir = FALSE, while all other parameters were set at their default values.

## 3. Results

### 3.1. Sustained Inhibition of ROCK Activity After Intravitreous Injection of BIRKI

**Figure 1:**
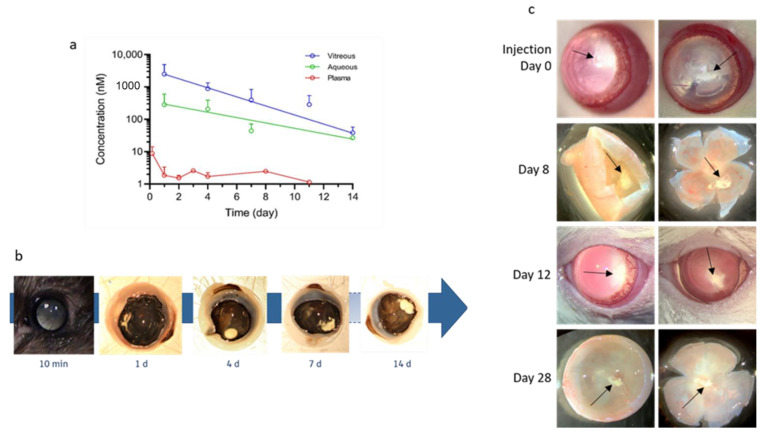
Evaluation of BIRKI release and duration in vivo. (**a**) BIRKI concentration (nM) measured in the vitreous, aqueous, and plasma of brown Norway rats 14 days after the intravitreal injection. BIRKI was found in the vitreous (blue) and the aqueous (green) up to day 14 p.a. It was also found in the plasma (red) for up to day 11 p.a. (**b**) Drug depot visible in white in the brown Norway eyes for 14 days. Here for 10 min, 1, 4, 7, and 14 days after the injection. One eye is shown as a representant. (**c**) Right after the injection in Goto-Kakizaki rats, BIRKI is visible in the vitreous as a white substance (arrow). In dissected eyes 8 days after the injection, the compound is still largely present over the retina. After 12 days, we also observed in vivo the presence of BIRKI in the posterior segment. Finally, dissection at day 28 showed that the BIRKI compound is, to a lesser extent, still present in the vitreous.

When formulated in a polysorbate 80/NaCl suspension, BIRKI forms a depot that allows a sustained exposure of the active compound for at least 14 days, as shown by the in vivo pharmacokinetic study (Figure 1a). After injection into the rat eye, BIRKI was detected mostly in the vitreous but also in the aqueous humor up to day 14. The compound depot was visible in the vitreous at 14 days (Figure 1b) and at 28 days post-injection, suggesting that it could last even longer (Figure 1c).

**Figure 2:**
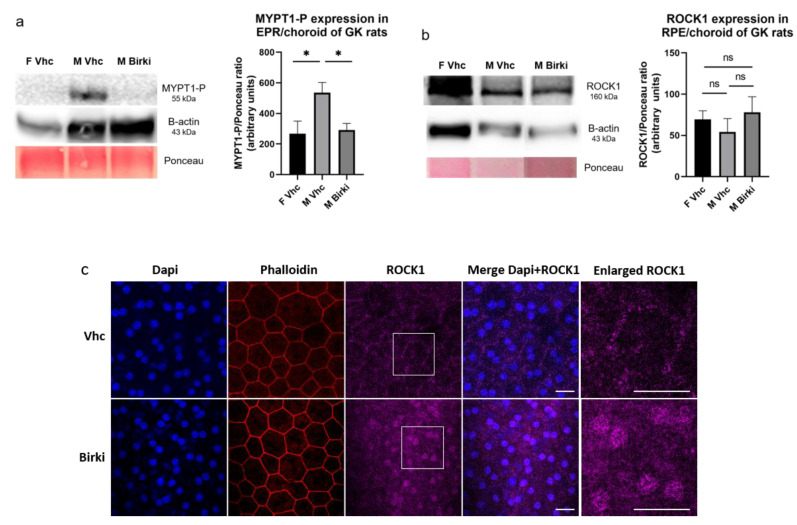
ROCK1 inhibition and relocalization: Western blots on RPE/choroid complex of vehicle or BIRKI-treated diabetic rats. (**a**) Western Blots showed a decrease in ROCK activity through the expression of phosphorylated MYPT1 8 days after the injection. Diabetic male GK rats treated with vehicle showed a higher phosphorylation rate of MYPT1 compared to non-diabetic female rats also treated with vehicle. Diabetic males treated with BIRKI showed a significant reduction in ROCK activity compared to vehicle-treated ones. Quantification was performed using Ponceau since actin might be altered in diabetic rats (Statistical significance was evaluated using the Kruskal–Wallis test, followed by the Dunn’s multiple comparison post-test, *n* = 6 F Vhc, *n* = 8 M Vhc and *n* = 11 M BIRKI, * means *p* < 0.05). (**b**) Western blots of ROCK1 protein expression. ROCK inhibition with BIRKI treatment did not modify ROCK1 protein expression in any group. Quantification was performed using Ponceau since actin might be altered in diabetic rats (Statistical significance was evaluated using the Kruskal–Wallis test, followed by the Dunn’s multiple comparison post-test, *n* = 3 F Vhc, *n* = 5 M Vhc and *n* = 9 M BIRKI). (**c**) RPE flat mounts stained with phalloidin (red), DAPI (blue), and ROCK1 (purple). In non-treated diabetic rats, ROCK1 is recruited at the cytoplasmic membrane, co-localizing with F-actin (red). In BIRKI-treated rats, inhibition of ROCK1 led to its relocation to the cytoplasm and nucleus, as shown on magnification. Scale bar represents 10 µm.

The compound released from the formulation efficiently inhibited ROCK activity in the retina of diabetic rats compared to vehicle-treated rats 8 days after injection, as shown by a significant reduction in phosphorylated MYPT1, a target commonly used to evaluate ROCK activity (Figure 2a). This demonstrates the efficacy of the treatment, although notably, ROCK1 protein expression remained unchanged, as shown in Figure 2b. ROCK1 immunolocalization further confirmed that BIRKI efficiently inhibited ROCK1 activation in RPE cells at 28 days, as shown by the change in its cellular distribution from the cell membrane in diabetic animals to relocation to the cytoplasm and the nuclei in treated rats (Figure 2c). Importantly, intraocular BIRKI did not influence the glycemic control of GK rats. Accordingly, no change was observed in HbA1C in GK diabetic rats after BIRKI injection (Supplementary Figure S1), although low and transient levels of BIRKI were measured in the plasma of treated rats (Figure 1a).

### 3.2. No Functional or Structural Damage Occurred Secondary to BIRKI Injection in the Rat Eye

**Figure 3:**
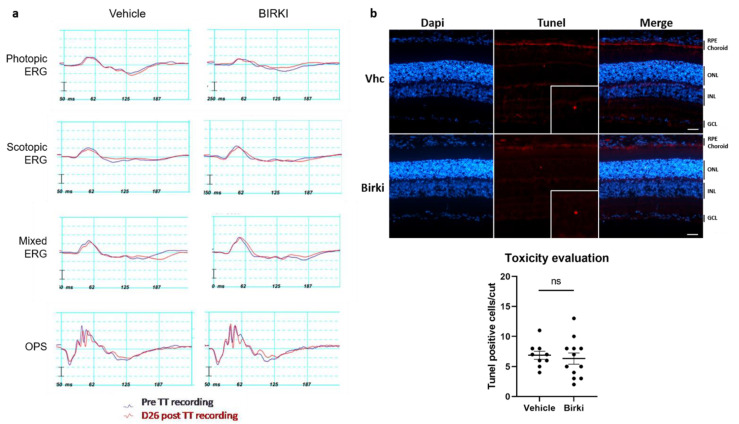
Intravitreal BIRKI injection does not alter retinal function in 14-month-old GK rats. There is no difference between vehicle-treated rats and BIRKI-treated rats in electroretinographic recordings (*n* = 3, 6 eyes per group). (**a**) Representative electrical ERG responses of vehicle (left) vs. BIRKI (right) treated rats, before (purple) and 26 days after (red) intravitreal injection. Recordings were performed with the Ganzfeld VisioSystem device (Siem Biomedicale, Nîmes, France), sending achromatic light flashes. For scotopic electroretinograms, representing essentially rod-driven responses, rats were dark-adapted overnight, then received flash intensities that ranged from 0.0003 to 10 cd s/m². For photopic electroretinograms, representing essentially cone-driven responses, rats were light-adapted for 5 min, and 10 cd s/m² flashes were sent. In both conditions, 5 responses were averaged. Mixed responses refer to whole retinal responses (coming from both cone and rod-driven pathways). Negative a-waves amplitudes were measured in a blind way from baseline to trough bottom. Positive b waves amplitudes were measured in a blind way also, from the b-wave trough bottom to its peak. Implicit times of a and b waves were measured from the time of stimulus to peaks. (**b**) TUNEL staining on cryosections. Apoptotic cells are stained in red while vessels are visible in green through FITC-dextran. Nuclei are in blue. Very few apoptotic cells are detectable on both BIRKI and control rat retinas 28 days after treatment. They are exclusively in the outer nuclear layer, as seen in the x4 magnification box. Scale bar represents 20 µm. ONL: outer nuclear layer, INL: inner nuclear layer, GCL: ganglion cell layer. After counting, there is no difference in TUNEL staining between BIRKI and control rats (Mann–Whitney statistical test *n* = 6, ns means *p* = 0.6119).

To evaluate any potential tolerability issues after intraocular BIRKI injection, electroretinography (ERG) was performed on 14-month-old GK rats, 8 days before the injection of either vehicle or BIRKI (as baseline recording, purple lines) and 26 days after the injection (red lines) (Figure 3a). Rats were sacrificed two days after the last recording for the TUNEL assay. No changes were observed in scotopic electroretinograms representing essentially rod-driven responses, in photopic electroretinograms representing essentially cone-driven responses, or in mixed responses (no significant changes in amplitude or implicit time between vehicle and BIRKI-treated rats were observed for each stimulus intensity, from 0.0003 to 10 cd.s/m^2^, graphs not shown, 0,13 < *p*-values < 1). In addition, no changes were observed in both a and b-wave amplitudes and in the implicit times between pre- and post-injection.

Quantification of TUNEL positive cells, performed on retina sections 28 days after the treatment, showed a similar low number of TUNEL positive cells in the outer nuclear layer of retina from GK rats treated with either vehicle or BIRKI (Figure 3b). Altogether, these results indicate that BIRKI in a depot formulation did not induce observable functional damage or retinal cell death and was generally considered safe and well-tolerated, thus allowing for the assessment of retinal effects in rats.

### 3.3. One Single Injection of BIRKI Significantly Reduced Retinal Hypoxia and Capillary Constriction in GK Rats at 28 Days

**Figure 4:**
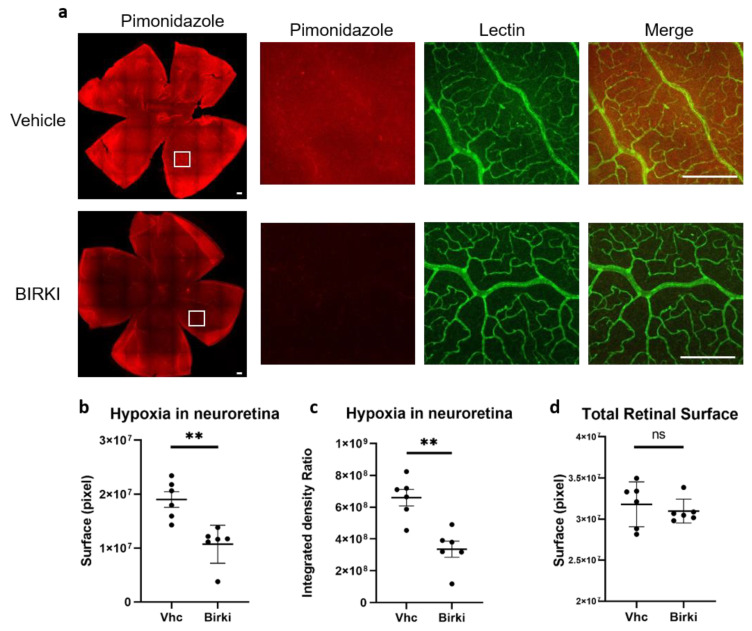
Effects of BIRKI on retinal hypoxia 28 days after treatment. (**a**) Hypoxic areas are stained in red with the pimonidazole probe, and vessels are stained in green with FITC-lectin on flat-mounted RPE. In vehicle diabetic rats, hypoxic areas also showed constricted and irregular capillaries, as shown in magnification. Hypoxic areas are smaller and less frequent in BIRKI-treated rats, and vessels are more regular. Scale bar represents 200 pixels. (**b**,**c**) Quantification of both intensity and surface of hypoxic staining zones was performed, showing a significant reduction in hypoxia in BIRKI-treated rats’ retinas compared to vehicle-treated. (Mann–Whitney test, *n* = 6 eyes, ** means *p* < 0.005). (**d**) We also checked that quantification was performed on same-surface retinas (Mann–Whitney test, *n* = 6 eyes, ns means *p* = 0.132). All the retinas have the same surface, allowing comparison.

**Figure 5:**
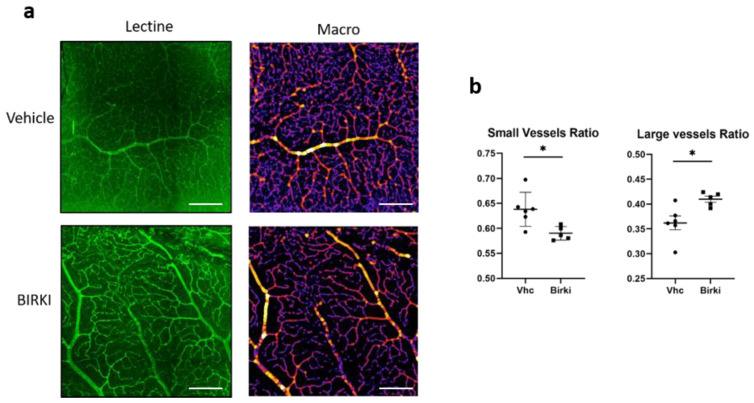
Effects of BIRKI on retinal vessel dilation. (**a**) Staining of the retinal capillary network was performed on retina flat mounts with FITC-lectin (in green). Vessels walls were detected automatically with a dedicated macro and classified (large vessels are represented in yellow while small vessels are purple, fake colors). Scale bar represents 200 pixels. (**b**) Quantification of the surface covered by small and large vessels with a dedicated macro. The retinal surface covered by small vessels was reduced in BIRKI-treated rats to the profit of larger vessels (Mann–Whitney test, *n* = 6 eyes, * means *p* < 0.05). 8–10 images per rat were randomly extracted from the previous mosaics in order to represent the whole flat mount with central and peripheral areas.

Retinal hypoxia, a consequence of reduced perfusion, was quantified 28 days after BIRKI or vehicle injection using pimonidazole, a probe classically used to detect hypoxic areas. As shown in Figure 4, there was a significant reduction in both surface area and intensity of retinal hypoxia after BIRKI treatment as compared to vehicle (*p* < 0.005. *n* = 6 eyes), indicating that BIRKI treatment reduced retinal hypoxia. The density and caliber of vessels stained with lectin appeared larger in areas with low pimonidazole staining (Figure 4a), suggesting the BIRKI effect on hypoxia could result from capillary dilation. Indeed, when BIRKI-induced vasodilation was quantified on lectin-stained vessels on flat-mounted retinae with a dedicated macro, we observed a shift of vessel size toward larger caliber capillaries in the eyes of BIRKI-treated animals (*p* < 0.05) (Figure 5)

### 3.4. BIRKI Reduced Retinal Vascular Leakage and Improved the RPE Morphology at 28 Days

**Figure 6:**
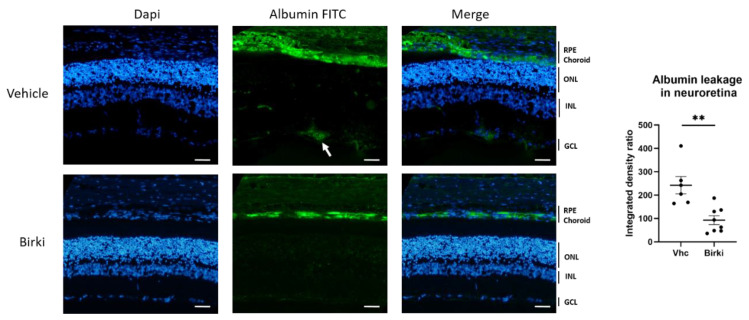
Effects of BIRKI on barrier leakage. Retinal leakage is visible through FITC-Albumin staining in green. Typical retinal barrier breakdown is visible in control diabetic rats (white arrow), while very few leakages were observed in BIRKI-treated rats 28 days after the injection. Scale bar represents 20 µm. ONL: outer nuclear layer, INL: inner nuclear layer, GCL: ganglion cell layer. Leakage quantification was performed by measuring the amount of fluorescence in both groups. There was a significant reduction in albumin leakage in the inner retina in BIRKI-treated rats compared to controls (Mann–Whitney statistical test, *n* = 6, ** means *p* < 0.005).

**Figure 7:**
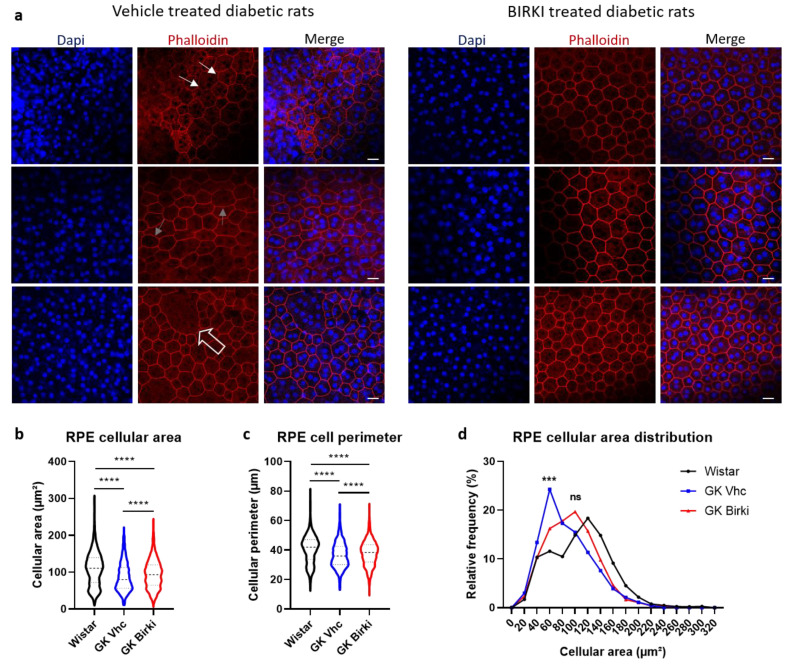
Evaluation of RPE morphology. (**a**) RPE flat mounts stained with DAPI (blue) and phalloidin (red) to observe the F-actin network. In diabetic rats, there are altered tight junctions (white plain arrows) and stress fibers, less present in BIRKI-treated rats. Giant RPE cells (syncytia showed by empty arrow) were also present in control rats, surrounded by smaller cells. We also observed rounded shape RPE cells (gray arrows) in control rats instead of hexagonal shape as seen in BIRKI rats. Scale bar represents 10µm. (**b**,**c**) Cellular area and cellular perimeter were analyzed on RPE flat mounts stained with phalloidin. RPE of GK rats showed a significantly reduced cellular surface and perimeter compared to Wistar rats. BIRKI-treated rats presented a significant increase but without a full recovery in both perimeter and cell area (Statistical significance was evaluated using the Kruskal–Wallis test, followed by the Dunn’s multiple comparison post-test, **** means *p* < 0.0001). (**d**) Represents the cellular distribution according to the area in µm². In diabetic rats, the shift toward smaller cells is visible in blue, while in BIRKI RPE rats in red, cells were bigger and the distribution relatively close to the Wistar ones in black. A chi-square test was used to compare cellular distribution. Wistar and diabetic vehicle-treated rats presented a significant difference in distribution. While Wistar and diabetic BIRKI-treated rats presented no difference between their cellular area distribution (statistical chi-square test with expected distribution was used, *** means *p* = 0.0009, ns means *p* = 0.0666). A total of six eyes per group, from 8 to 20 images per eye, and 60 to 120 RPE cells per image were quantified for (**b**–**d**).

Vascular leakage of macromolecules that contributes to retinal edema was significantly reduced 28 days after BIRKI injection. Albumin leakage around vessels and in the inner retinal layer was significantly reduced in BIRKI-treated rat retina as compared to vehicle-treated animals (*p* < 0.005) (Figure 6). Because RPE integrity is essential to maintain the outer retina health and prevent macular edema, we analyzed the effect of BIRKI on the RPE morphology since ROCK1 exerts an essential function in the maintenance of cytoskeleton integrity in this highly polarized epithelium. In diabetic rats, RPE cells show alteration of cell junctions, irregular shapes, and abnormal cell size (Figure 7a). All these morphological alterations were reduced 28 days after one single BIRKI injection as quantified by the perimeter of cells, the area of cells, and the size distribution (Figure 7b–d). Moreover, BIRKI treatment significantly normalized RPE morphology and shifted the cellular area distribution toward the morphology observed in non-diabetic Wistar rats (Figure 7d), demonstrating a positive effect of BIRKI on RPE cytoskeleton.

### 3.5. Transcriptomic Effects of BIRKI in Neuroretina and RPE/Choroid Complex at 28 Days After Injection

**Figure 8:**
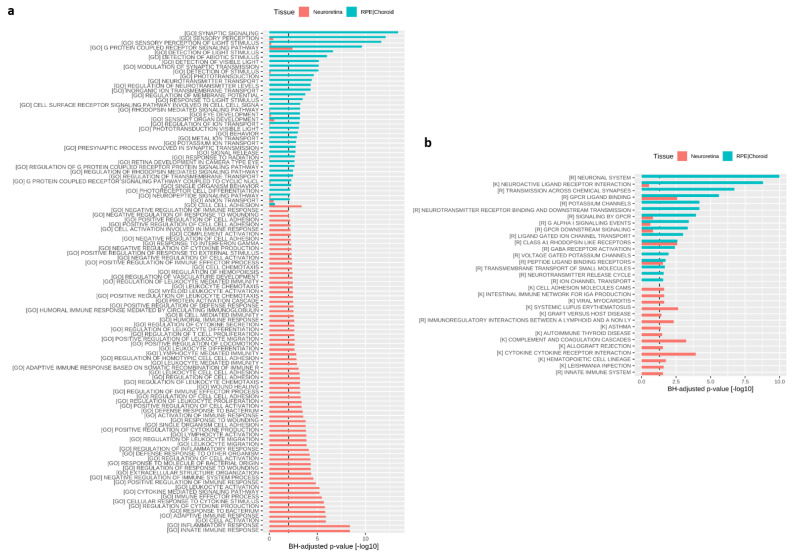
Transcriptomics effects of BIRKI on neuroretina and RPE/choroid complex. (**a**) GO_pathways regulated in treated neuroretinas (in orange) and RPE/choroid complexes (in blue) 28 days after the BIRKI injection. Shown are significantly regulated pathways (BH adjusted *p*-value < 0.01) in at least one of the tissues. (GO): GO biological processes. (**b**) reactome and KEGG pathways significantly regulated (BH adjusted *p*-value < 0.05) in at least one of the tissues (neuroretinas in orange and RPE/choroid complexes in blue). (K): KEGG, (R): REACTOME.

To investigate the effects of treatment on gene expression, we further performed RNA sequencing on the retina and RPE/choroid samples of vehicle as compared to BIRKI-treated GK rats (*n* treated = 4; *n* control = 6). Only a limited number of genes were differentially regulated (Benjamini Hochberg (BH)-adjusted *p*-value < 0.05) in either of the tissues between vehicle and BIRKI injected rats. We therefore performed fold-change-based gene set enrichment analysis (GAGE), which showed that BIRKI regulated distinct pathways in the neural retina as compared to the RPE/choroid (Figure 8). In the RPE/choroid, the gene ontology biological process (GO BP) pathways wound healing, regulation of transmembrane transport, regulation of membrane potential, ion transport, potassium-ion transport, and inorganic ion transmembrane transport pathways were affected, suggesting an effect on RPE cell integrity. In addition, a number of GO BP pathways involved in synaptic transmission and neuropeptides were regulated, which refer to the sympathetic neural control of the choroidal vasculature (synaptic signaling, regulation of neurotransmitter levels, pre-synaptic process involved in synaptic transmission, neurotransmitter transport, neuropeptide signaling pathway, modulation of synaptic transmission). We further observed the regulation of pathways related to visual function. In contrast to RPE/choroid, in the neural retina, regulated pathways were predominantly involved in immune response and inflammation, or response to wound healing and coagulation (Figure 8a). Evaluation of reactome and KEGG gene sets provided similar observations with respect to biological processes affected by BIRKI treatment (Figure 8b).

## 4. Discussion

ROCK inhibition appears to be a promising therapeutic concept to target various pathogenic mechanisms in the diabetic retina. In vitro, ROCK1 is activated by high glucose in human retinal capillary endothelial cells, inducing their proliferation [21], while in a rhesus macaque choroid-retinal endothelial cell line, it disrupts the tight junction complex and increases permeability [22]. In rodents with streptozotocin-induced type 1 diabetes, ROCK1 activation contributed to retinal vascular barrier disruption [23]. In the type 2 diabetic GK rat model, ROCK1 is activated at the membrane of smooth muscle cells and endothelial cells in areas of constricted capillaries and vessels and is reported to contribute to lumen closure through vascular constriction and endothelium blebs [19]. In addition, ROCK activation contributes to Müller glia damage [15] and pro-inflammatory signaling in the diabetic retina [14]. In RPE cells, ROCK activation contributes to fibrotic changes induced by transforming growth factor (TGF)-β [24] and to outer barrier disruption in the GK rat [19]. Interfering with ROCK activation could thus be effective in the treatment of diabetic macular edema, but also retinal ischemia and inflammation.

Several ROCK inhibitors have been developed to treat hypertension, stroke, or heart failure, and various side effects have been reported. Intravitreal injection lowers the risk of side effects compared to systemic administration due to the very limited systemic passage of the drug. However, the previously described ROCK inhibitors would require frequent administration due to their relatively short half-life in the vitreous chamber [25]. For instance, ripadusil, also called K-115 and developed to treat glaucoma and ocular hypertension [13], reduces macular edema by regulating the tight junction integrity in the retina [26]^.^ It also suppresses vasoconstriction, hyperpermeability, and edema by increasing blood flow one day after intravitreal injection in a retinal vein occlusion mouse model [27]. Another existing drug is fasudil, a non-specific ROCK inhibitor initially used in humans for the treatment of cerebral vasospasm [28]. Fasudil showed positive effects on reducing diabetic retinal leukocyte accumulation and endothelial damage [16]. We previously demonstrated that GK rats treated with three consecutive intravitreal injections of fasudil over 48 h presented improved retinal perfusion associated with decreased retinal hypoxia and restoration of the RPE barrier [19]. As shown in the present study, one single injection of BIRKI exerts at 28 days similar effects as the short-term effect of fasudil in the same animal model. The BIRKI suspension is able to form a depot in the vitreous that slowly dissolves but is still visible after a month. It is injectable through 30G needles and should cause only limited visual disturbance and floaters due to the formation of a depot, which could represent an advantage for clinical use, where the complete resorption of the product could indicate the right timing for re-injection. This remains a variable parameter with any injectable product into the vitreous. In addition, we do not observe any negative functional or morphological effects, measured by electroretinography and apoptotic marker indicating no tolerability issues. However, toxicology studies would be needed to ensure the long-term safety of BIRKI release into the eye.

To better understand and characterize the effects of BIRKI in the diabetic retina and in the RPE/choroid complex, a full transcriptomic analysis was performed in both tissues after 4 weeks of drug exposure. In the neural retina, ROCK inhibition mostly acts on the neurovascular unit, which is shown by transcriptional regulations of vascular, immune, and inflammatory pathways. Interestingly, ROCK inhibition does not interfere, at least at the transcriptional level, with major visual pathways in the neural retina, which is in agreement with the suitable retinal functional tolerability observed. Enrichment in cells that constitute the inner blood-retinal barrier could allow a more specific analysis of the effects of BIRKI on these structures. Interestingly, the effects of BIRKI observed on the outer RPE barrier are also corroborated by the transcriptomic analysis in the RPE/ choroid complex, demonstrating that the drug is reaching these deep retinal layers. Most of the differentially regulated pathways are related to choroid neural regulation. This regulation is essential to control the blood flow in the choroid, where the autonomous neural system regulates the proper oxygen and nutrient supply to the photoreceptor cells. In diabetes, autonomic neuropathy [29,30] could contribute to choroid and outer retina pathology [31,32,33,34]. Since ROCK inhibition was shown to promote nerve regeneration [35,36] and the vascular effect of ROCK to act at least in part through sympathetic nerve-mediated ROCK activation [37], further studies could explore the effect of ROCK inhibition on choroidal neuropathy in diabetic conditions.

In conclusion, this study confirms that chronic and local intraocular ROCK inhibition is effective in reducing retinal ischemia and restoring both inner and outer retinal barriers in rats in vivo. Furthermore, it does not cause functional retinal side effects. Yet, the translation to human disease is still to be confirmed. As convenient long-acting novel treatment options are particularly important for application in humans, further development of intravitreal sustained-release formulations of ROCK inhibitors is needed for patients with both macular edema and ischemia.

## Data Availability

Data can be provided upon reasonable request.

## Supplementary Materials

The following are available online at https://www.mdpi.com/article/10.3390/pharmaceutics13081105/s1, Figure S1: The diabetic status of every animal was defined by measurement of the plasma concentration of glycosylated hemoglobin (HbA1c).
