# Supplementary material for: Evaluation of an Intravitreal Rho-Associated Kinase Inhibitor Depot Formulation in a Rat Model of Diabetic Retinopathy
Source: Pharmaceutics. 2021 Jul 21;13(8):1105. doi: 10.3390/pharmaceutics13081105 (PMC8401380; doi:10.3390/pharmaceutics13081105)
Supplement: Supplementary file 1 [file pharmaceutics-13-01105-s001.zip › pharmaceutics-1263533-supplementary.pdf]

pharmaceutics

Supplementary Materials: Evaluation of an Intravitreal Rho-Associated Kinase Inhibitor Depot Formulation in a Rat Model of Diabetic Retinopathy

Cecile Lebon, Heike Neubauer, Marianne Berdugo, Kimberley Delaunay, Elke Markert, Kolja Becker, Katja S. Baum-Kroker, Jürgen Prestle, Holger Fuchs, Remko A. Bakker and Francine Behar-Cohen

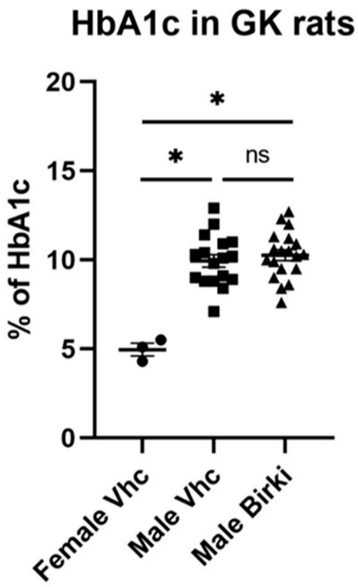

Figure S1. The diabetic status of every animal was defined by measurement of the plasma concentration of glycosylated hemoglobin (HbA1c). Mean of HbA1c is 4.96% for female GK rats (Average Blood Glucose: 96 mg/dL) and 9.94 and 10.26% for GK male treated with vehicle and BIRKI respectively (ABD: 239 and 248 mg/dL). Statistical significance was evaluated using the Kruskal-Wallis test, followed by the Dunn's multiple comparison post-test. * means $p < 0.05$
